# Effects of Multiday Exposure to Ozone on Airway Inflammation as Determined Using Sputum Induction

**DOI:** 10.1289/ehp.8341

**Published:** 2005-09-29

**Authors:** Jeffrey Ratto, Hofer Wong, Jane Liu, John Fahy, Homer Boushey, Colin Solomon, John Balmes

**Affiliations:** 1 Lung Biology Center,; 2 Division of Pulmonary and Critical Care Medicine, and; 3 Northern California Center for Occupational and Environmental Health, University of California San Francisco, San Francisco, California, USA

**Keywords:** airway inflammation, multiday exposure, ozone, proximal airways, sputum induction

## Abstract

Single short-term exposures to ozone are known to cause acute changes in pulmonary function and neutrophilic airway inflammation. The respiratory health effects of repeated exposures are not as well studied. Pulmonary function decrements are known to attenuate, but it is less clear how injury and inflammation are affected. Using sputum induction (SI) to sample respiratory tract lining fluid after single- and multiday exposures, we designed a study to test the hypothesis that neutrophils would increase after multiday exposure compared with single-day exposure. In a randomized, crossover design, 15 normal healthy subjects were exposed to O_3_ (0.2 ppm) under two conditions: for 4 hr for 1 day (1D) and for 4 hr for 4 consecutive days (4D). Pulmonary function testing was performed immediately before and after each 4-hr exposure. The SI was performed 18 hr after the end of the 1D and 4D conditions. The symptom and pulmonary function data followed a pattern seen in other multiday O_3_ exposure studies, with the greatest changes occurring on the second day. In contrast to previous studies using bronchoalveolar lavage, however, there was a significant increase in the percentage of neutrophils and a significant decrease in the percentage of macrophages after the 4D condition compared with the 1D condition. Given that SI likely samples proximal airways better than distal lung, these results add to the body of evidence that differential airway compartmental responses to O_3_ occur in humans and other species.

Ozone is an oxidant gas that is an important component of urban air pollution. It is generated from oxygen by photochemical reactions involving oxides of nitrogen and hydrocarbons produced by combustion. Environmental O_3_ standards and guidelines include those of the World Health Organization (WHO 2000) and European Union (European Communities 2002), 120 μg/m^3^ (0.06 ppm); and U.S. Environmental Protection Agency (U.S. EPA 2004), 0.08 ppm (167 μg/m^3^)—each as an average for an 8-hr period. These levels are exceeded in some regions; in Europe, O_3_ levels routinely exceeded 0.1 ppm during the summer of 2003 (Fiala et al. 2003), and in Mexico City 1-hr averages were as high as 0.102 ppm (Romieu et al. 2004).

The acute respiratory health effects of short-term exposures to O_3_ have been well characterized through controlled human exposure studies of healthy subjects using O_3_ concentrations from 0.2 to 0.6 ppm (Aris et al. 1993; Balmes et al. 1996; Koren et al. 1989; Seltzer et al. 1986). Single short-term exposures are known to cause acute changes in pulmonary function, such as increased specific airways resistance (SR_aw_) and decreased forced expiratory volume in 1 sec (FEV_1_) and forced vital capacity (FVC) (Aris et al. 1993; Balmes et al. 1996). In addition, indices of inflammation in respiratory tract lining fluid (RTLF), as measured in bronchoalveolar lavage (BAL) and sputum induction (SI), are increased by short-term exposures. The inflammatory response most consistently seen includes an increase in neutrophils (Aris et al. 1993; Koren et al. 1989; Seltzer et al. 1986). Other markers of inflammation that also are increased after O_3_ exposure include fibronectin, total protein, and cytokines such as interleukin (IL)-6 and IL-8 (Balmes et al. 1996; Koren et al. 1989).

The respiratory health effects of multiday exposure are not as well studied. Pulmonary function decrements are known to attenuate on the third and fourth day of multiday exposures (Christian et al. 1998; Hackney et al. 1977). It is less clear how injury and inflammation are affected by multiday exposures. A study from our laboratory showed attenuation of the inflammatory response in BAL in healthy subjects exposed to 0.2 ppm O_3_ for 4 hr on 4 consecutive days compared with a single-day exposure (Christian et al. 1998). A similar study by Jorres et al. (2000) used both BAL and endobronchial biopsies to assess airway inflammation. BAL fluid showed an increase in neutrophils after the single-day but not after the 4-day exposure. IL-6, IL-8, and total protein were increased after both exposure arms in the BAL fluid. The endobronchial biopsies, representing proximal airways, showed increased neutrophils after the 4-day exposure but not after the single-day exposure.

SI is less invasive and less technically difficult than bronchoscopy and has been used to obtain RTLF to assess airway inflammation after O_3_ exposure (Fahy et al. 1995; Hiltermann et al. 1995). It is not clear that samples of RTLF obtained by SI and BAL are interchangeable for measuring O_3_-induced airway inflammation (Arjomandi et al. 2005). SI and BAL may be primarily sampling different compartments of the airway, more proximal for SI and more distal for BAL (Alexis et al. 2001). Also, data from induced sputum may be more inherently variable than are data from BAL, making it more difficult to detect O_3_-induced inflammation (Arjomandi et al. 2005).

In this study we used SI to sample RTLF after single- and multiday exposures to assess the effect of repeated exposures to O_3_ on proximal airways. Our hypothesis was that neutrophils in induced sputum would increase as a function of multiday exposure compared with single-day exposure.

## Materials and Methods

### Design.

In this study we used a two-condition, single-blind, randomized, crossover design. Subjects completed an initial characterization session and subsequently two exposure conditions: exposure to O_3_ (0.2 ppm) for 4 hr for 1 day (1D) and exposure to O_3_ (0.2 ppm) for 4 hr for 4 consecutive days (4D; days 1–4: 4D-1, 4D-2, 4D-3, 4D-4). To assess airway inflammation, we conducted SI 18 hr after the exposure in the 1D condition and 18 hr after the fourth exposure of the 4D condition. We assessed lung function response by spirometry and by measuring airway resistance preexposure, postexposure, and pre-SI. The two exposure conditions were separated by a minimum of 3 weeks.

### Subjects.

Fifteen healthy nonsmoking subjects (seven male, eight female; mean ± SD age = 24 ± 2.9 years) participated. The study protocol was approved by the Committee on Human Research of the University of California San Francisco; all applicable requirements for ethical human research were met; all subjects gave written informed consent before enrollment. Subjects completed a medical history questionnaire and were characterized by physical, spirometric, and nonspecific airway reactivity measurements (Table 1). Subjects had no previous or current lung disease or other serious disease.

### Pulmonary function.

We performed spirometry using a rolling seal spirometer (Ohio 840, Ohio Medical, Dayton, OH) according to criteria of the American Thoracic Society (American Thoracic Society 1987). SR_aw_ was calculated as the mean of five measurements of airway resistance and thoracic gas volume in a constant-volume variable-pressure whole-body plethysmograph (Warren E. Collins Inc., Braintree, MA) measured every 30 sec for 2.5 min. We conducted spirometry and plethysmography for subject characterization, immediately pre- and postexposure, and pre-SI.

### Nonspecific airway reactivity.

For subject characterization, we determined nonspecific airway reactivity using increasing doses of methacholine to observe a 20% decrease in FEV_1_.

### Exposure.

We conducted all exposures with the subject in a stainless steel and glass chamber (8 × 8 × 8 ft) to control for environmental air temperature and humidity. The chamber air supply was passed through two filters in series, a charcoal filter and a Purafil filter (Purafil Inc., Atlanta, GA), and chamber temperature and relative humidity were continuously monitored. O_3_ was generated by passing 100% oxygen through an ozonator (Welsbach no. T-408; Ozone Engineering, El Sobrante, CA), and the concentration was targeted to 0.2 ppm as measured by an ultraviolet O_3_ analyzer (model 1003; Dasibi Environmental Corp., Glendale, CA). O_3_ exposures were conducted using a mouth-breathing facemask (Hans Rudolph Inc., Kansas City, MO) fitted with a nonrebreathing valve (Hans Rudolph 2600). There were no significant differences between any of the exposure conditions, O_3_ concentration, temperature, and relative humidity, between the 1D and 4D conditions and within the 4D condition (Table 2). Exposures were of 4-hr duration.

### Exercise.

During the exposure, subjects performed intermittent exercise (30 min of each 60 min) on a cycle ergometer (Corvial 400; Quinton Instruments Co., Seattle, WA). The work level selected was based on the workload required to achieve and maintain a calculated minute ventilation of 25 L/min × body surface area. During each 30-min exercise period, minute ventilation was measured at 10 and 20 min using a section of corrugated tubing placed between the valve on the face-mask and a Fleisch pneumotachograph (General Medical Corp., West Sacramento, CA); flow signals recorded from the pneumotachograph were amplified (Validyne Engineering Corp., Northridge, CA) to yield volume signals and then recorded on a visicorder (Honeywell 1858; Honeywell Test Instruments Division, Denver, CO). There was a statistically significant decrease of minute ventilation in 4D-4 compared with both 1D and 4D-1 [median (25–75% range); 46.3 (42.0–51.4) L/min vs. 48.3 (45.0–53.8) L/min and 48.6 (43.0–57.8) L/min; *p* = 0.029 and *p* = 0.031, respectively]. There were no other significant differences between conditions or within the 4D condition.

### Sputum induction.

All subjects were pre-treated with 180 μg albuterol administered by metered-dose inhaler and then inhaled nebulized sterile 3% saline for 20 min from an ultrasonic nebulizer [DeVilbiss Ultra-Neb 99; DeVilbiss Air Power Co., Jackson, TN; this nebulizer generates particles of a mean mass median diameter of 3.5 μm and has an output of 5.9 mL/min]. Subjects were encouraged to cough airway secretions into a plastic container throughout the procedure. At 4-min intervals, subjects were instructed to cough airway secretions and saliva into the plastic container, and a peak flow measurement was obtained. SI was performed 18 hr postexposure.

### Sputum processing.

The volume of the induced sputum sample was determined and then mixed with an equal volume of 0.1% dithiothreitol (Sputalysin 10%; Behring Diagnostics Inc., Somerville, NJ) for homogenization. The sample was then mixed gently by vortex mixer and placed in a shaking water bath at 37°C for 15 min to ensure complete homogenization. The sample was removed from the water bath periodically for further brief gentle vortex mixing. One milliliter of the homogenized sputum sample was aliquoted for cell counts, and the remainder of the sample was centrifuged at 1,037 × *g* for 5 min. The supernatant was aspirated and stored at −70°C for subsequent protein analysis.

### Cell counts.

Ten microliters of homogenized induced sputum was used to determine the total leukocyte count using a standard hemacytometer. For cell differentiation, 250-μL aliquots of homogenized induced sputum were spun in a cytocentrifuge (model 7 Cytospin; Shandon Scientific, Pittsburgh, PA) onto glass slides that were then stained (May Grunwald Giemsa stain). Differential cell counts included macrophages, lymphocytes, neutrophils, and eosinophils (each expressed as a percentage of total leukocytes). One investigator, blinded to the subject’s exposure condition, counted at least 400 cells.

### Protein assays.

We measured total protein using the Bradford method, with reagents from Bio-Rad Laboratories (Hercules, CA). Lactate dehydrogenase (LDH) was measured using a spectrophotometer and a LDH reagent (LD-L; Sigma-Aldrich, St. Louis, MO). Fibrinogen was measured by enyzme-linked immunosorbent assay (ELISA) with reagents from Sigma-Aldrich. IL-6 and IL-8 levels were measured using immunoassays (ELISA, Quantikine; R&D Systems, Minneapolis, MN) with lower limits of detection of 0.09 and 18.0 pg/mL, respectively. Detailed descriptions of our methods for fluid-phase measurements of soluble proteins in induced sputum have been published previously (Fahy et al. 1993).

### Symptoms.

The symptom score was based on a questionnaire with a 5-point scale [0 (not noticeable), 1 (minimal), 2 (mild), 3 (moderate), and 4 (severe)] administered immediately before and after exposure. Symptoms rated were chest discomfort, chest pressure, cough, sputum production, shortness of breath, and wheezing.

### Statistical analysis.

We compared data from the exposure environments (O_3_ concentration, temperature, humidity) using the paired *t*-test. Because most of the data for the dependent variables (cell counts, protein concentrations, spirometry) did not fit a normal distribution, both within- and between-condition paired comparisons were conducted using the Wilcoxon signed rank test. We considered a *p*-value of < 0.05 significant. Post hoc power calculations indicated that the sample size of 15 provided 0.78 power at α = 0.10 and 0.66 power at α = 0.05 to detect the observed difference in neutrophils between the 1D and 4D conditions.

## Results

### Cells and proteins.

**Figure 1:**
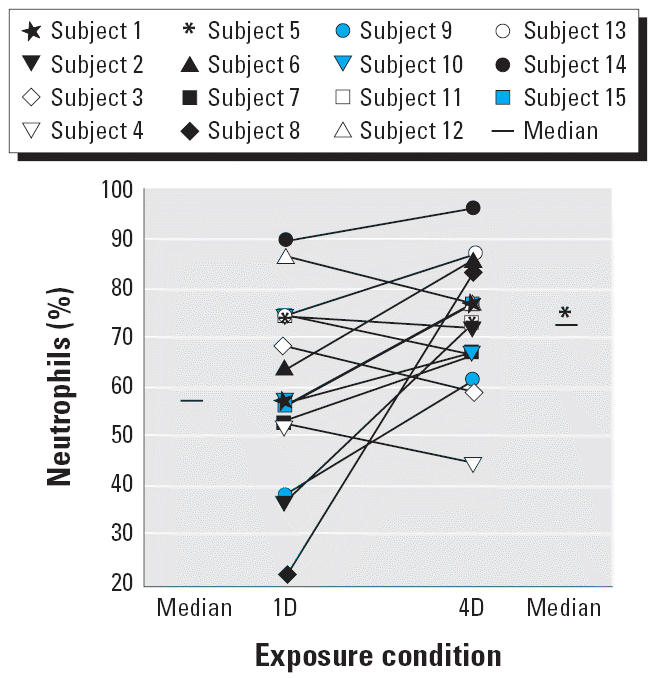
Individual data and group median values for neutrophil percentage for the 1D and 4D conditions. *p = 0.02 versus 1D neutrophils (%) (Wilcoxon signed-rank test).

For the cell data, we compared the 4D condition with the 1D condition and found a significant increase in the percentage of neutrophils and a significant decrease in the percentage of macrophages (Table 3; Figure 1). There were no other significant differences in any other cell percentages. We also found no significant differences for total protein concentration, LDH, fibrinogen, IL-6, or IL-8 when we compared the 1D condition with the 4D condition (Table 3).

### Pulmonary function.

**Figure 2:**
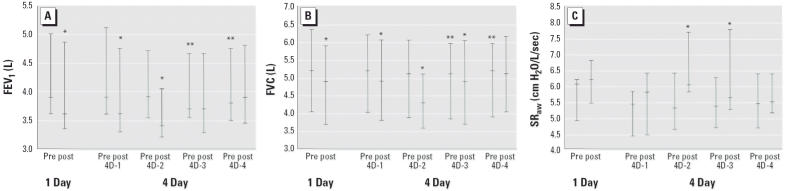
FEV_1_ (*A*), FVC (*B*), and SR_aw_ (*C*) preexposure and postexposure values in the 1D and 4D (4D-1, 4D-2, 4D-3, 4D-4) O_3_ conditions. Values are median (25–75% range). Abbreviations: Pre, preexposure; post, postexposure. For FEV_1_: *significant difference between preexposure and postexposure for 1D (*p* = 0.004), 4D-1 (*p* = 0.005), and 4D-2 (*p* = 0.001); **significant difference for preexposure 1D compared with both preexposure 4D-3 (*p* = 0.006) and preexposure 4D-4 (*p* = 0.004). For FVC: *significant difference between preexposure and postexposure for 1D (*p* = 0.002) and 4D-1 (*p* = 0.004), 4D-2 (*p* = 0.001), and 4D-3 (*p* = 0.026); **significant difference for preexposure 4D-2 compared with both preexposure 4D-3 (*p* = 0.009) and preexposure 4D-4 (*p* = 0.049). For SR_aw_: *significant difference between preexposure and postexposure for 1D (*p* = 0.016) and on 4D-2 (*p* = 0.004) and 4D-3 (*p* = 0.006). All *p*-values are from Wilcoxon signed-rank test.

There were some small but statistically significant differences in preexposure values for FEV_1_ and FVC between the 1D and 4D conditions and within the 4D condition (Figure 2). There were no statistically significant differences in the values for the preexposure SR_aw_ either between the conditions or within the 4D condition (Figure 2). For FEV_1_ and FVC there were significant decreases from preexposure to postexposure in the 1D condition (Figure 2). FEV_1_ and FVC also decreased significantly on 4D-1 and 4D-2 (Figure 2). 4D-3 FVC had a significant decrease and 4D-3 FEV_1_ had a decrease that was not significant (Figure 2). The difference from preexposure to postexposure was not statistically significant for either FEV_1_ or FVC on 4D-4 (Figure 2). Comparing the pre-exposure SR_aw_ with postexposure measurement, we observed statistically significant increases for 1D and for 4D-2 and 4D-3 (Figure 2).

### Symptoms.

Symptom ratings had no significant difference for the preexposure values between conditions or within the 4D condition. The difference between the preexposure and postexposure score was significant for the 1D condition, and all 4 days of the 4D condition. The symptom change decreased from 4D-2 through 4D-4 in the 4D condition. The change in symptom rating [median (25–75% range)] for the 1D exposure was 8 (3–11.5), and for the 4D exposure the changes were 5 (2–8) for 4D-1, 7 (5.5–10) for 4D-2, 3 (2–4) for 4D-3, and 0 (0–1) for 4D-4. The 4D-2 change was significantly different from the 4D-3 and 4D-4 change, and the 4D-3 change was significantly different from the 4D-4 change.

## Discussion

The results of this experiment confirmed our hypothesis that neutrophilic inflammation would increase in induced sputum samples after a multiday exposure to O_3_ compared with a single-day exposure. Neutrophils were increased as a percentage of total leukocytes in the multiday condition. Compared with a filtered air exposure condition from a previous study (Fahy et al. 1995), the neutrophil percentage (mean; expressed as a percentage of nonsquamous cells) was elevated in the 1D O_3_ condition, and to a larger extent in the 4D O_3_ condition (filtered air, 51.0%; current 1D O_3_, 55.3%; current 4D, 69.8%). This indicates that our 1D exposure to O_3_ caused neutrophil-associated inflammation, with increased inflammation after 4 days of exposure. The macrophage percentage was decreased with 4D exposure, although this was mostly due to the increase in neutrophils. There were no significant changes in any of the other measured cell and protein end points.

The symptom and pulmonary function data followed a pattern seen in other multiday O_3_ exposure studies. The greatest changes in pulmonary function values (decreases in FEV_1_ and FVC and an increase in SR_aw_) occurred on day 2 of multiday exposure in both the present and previous studies (Hackney et al. 1977; Christian et al. 1998; Jorres et al. 2000). The largest increase in respiratory symptoms also occurred on the second day, in both the present and previous studies (Christian et al. 1998). By day 4 of the multiday O_3_ exposure, there was almost complete attenuation of both the pulmonary function and symptom responses to O_3_. However, preexposure FEV_1_ was significantly lower on days 3 and 4 compared with the preexposure value on day 1, suggesting that full recovery from the effect of the previous day’s exposure had not occurred.

The most surprising finding of the present study is that there was no attenuation of the neutrophil recruitment to the airways with the multiday O_3_ exposure arm. This finding is in contrast to those of previous studies by our group and others in which attenuation of this response using BAL to sample RTLF has been observed (Devlin et al. 1997; Christian et al. 1998; Jorres et al. 2000). It is consistent, however, with the increased recruitment of neutrophils to proximal airway tissue demonstrated in endobronchial biopsy samples after multiday exposure to O_3_ (Jorres et al. 2000). The increased neutrophils that we observed after multiday exposure to O_3_ in this study could be due to multiple factors, but a differential response between proximal and distal airway compartments must be considered.

Although the evidence on which airway compartments are sampled by SI is somewhat conflicting, it is believed to sample proximal airways preferentially compared with BAL, which more preferentially samples distal airways and alveoli (Alexis et al. 2001; Gershman et al. 1999). BAL samples only distinct segments of the lung distal to the bronchus into which the bronchoscope is wedged (Kelly et al. 1988). In contrast, SI probably provides a more representative sample of proximal airways, although with prolonged induction the distal parts can also be sampled as evident from increased numbers of macrophages from the alveolar compartment (Gershman et al. 1999; Holz et al. 1998). Thus, it is interesting to note that after single exposures to O_3_, both SI and BAL show increased neutrophils, consistent with both proximal and distal airway inflammatory responses. Differences in BAL compared with induced sputum in multiday studies could be due to differences in the way O_3_ is absorbed by, antioxidant defenses of, or the nature of the inflammatory responses in proximal and distal airway compartments, or the possibility that neutrophils recruited to distal airways after O_3_ exposure migrate to more proximal airways. Animal studies have shown differences in deposition of O_3_ and subsequent airway injury throughout the airways (Plopper et al. 1998). Dose-dependent compartmental variations in antioxidant enzyme activity have been demonstrated in mice, rats, and rhesus monkeys (Duan et al. 1996a, 1996b; Plopper et al. 1998). Differential O_3_ deposition or amount of antioxidant enzymes present in various airway compartments could also occur in humans. This could lead to differences in the inflammatory response to O_3_ in different regions of the airway with multiday exposures, that is, attenuation of neutrophil recruitment in distal areas and increased recruitment in proximal areas.

Potential limitations of our study must be considered. First, a filtered-air exposure control condition would have allowed direct determination of the independent effects of the 1D O_3_ and 4D O_3_ conditions. Second, the number of subjects (*n* = 15) is a function of this being a controlled human exposure study involving a complicated protocol. A larger number of subjects may have changed our results, although multiple studies of the airway inflammatory effects of O_3_ exposure have detected exposure-related differences in airway inflammatory responses with a similar sample size (Christian et al. 1998; Fahy et al. 1995; Jorres et al. 2000). Third, the time point for sampling of RTLF (18 hr postexposure) that we selected could be questioned as not being at the peak of O_3_-induced neutrophil recruitment (Schelegle et al. 1991). We chose 18 hr postexposure for RTLF sampling because inflammation is still present and our laboratory has considerable experience with analysis of samples obtained at this time.

In conclusion, the major finding of this study is the lack of attenuation of neutrophilic inflammation in induced sputum samples after a multiday exposure to O_3_ compared with a single-day exposure. This result stands in marked contrast to several studies, including one from our group in which attenuation of neutrophil recruitment was observed when BAL was used to sample airway lining fluid. These contrasting findings add to the body of evidence that differential airway compartmental responses to O_3_ occur in humans and other species. Such differential responses may have importance for understanding the clinical impact of ambient O_3_ exposures on susceptible subgroups (e.g., persons with asthma). Thus, further research on potential mechanisms underlying compartmental responses to O_3_-induced oxidative stress seems warranted.

## Figures and Tables

**Table 1:** Individual subject characteristics.

				FEV_1_		FVC			
Subject	Sex	Age (years)	Height (cm)	Liter	% predicted	Liter	% predicted	SR_aw_ (cm H_2_O/L/sec)	PC_20_ (mg/mL)
01	M	31	167	3.45	91	3.90	83	6.9	> 64
02	F	20	153	3.65	125	4.05	113	4.4	> 64
03	M	24	180	5.45	123	6.30	114	5.3	> 64
04	M	27	174	4.20	100	5.20	99	6.7	> 64
05	F	23	167	3.90	117	4.15	100	3.9	10.72
06	M	23	189	5.70	118	7.00	113	5.9	14.59
07	F	27	168	3.35	102	3.85	94	5.0	> 80
08	F	26	168	3.55	108	4.35	105	5.4	5.61
09	F	22	168	3.55	105	4.00	95	3.5	15.27
10	F	25	171	3.90	113	5.40	125	5.9	> 80
11	M	28	186	4.70	103	7.75	132	7.1	4.66
12	F	23	173	3.75	106	4.15	94	5.1	12.6
13	F	22	166	4.25	127	5.25	127	6.0	> 80
14	M	22	187	5.45	113	6.25	102	5.1	10.50
15	M	24	185	5.55	119	6.95	117	5.9	> 80
Mean	7 M 8 F	24.5	173.3	4.29	111	5.24	108	5.48	NA
SD		2.9	10.0	0.85	10.2	1.32	14.0	1.05	NA

Abbreviations: F, female; M, male; NA, not applicable; PC_20_, provocative concentration of methacholine producing a > 20% decrease in FEV_1_.

**Table 2:** Exposure characteristics (mean ± SD).

Exposure characteristic	1-day exposure	4-day exposure
O_3_ (ppm)	0.201 ± 0.008	0.204 ± 0.09
Temperature (°C)	19.9 ± 0.29	19.8 ± 0.31
Relative humidity (%)	52.0 ± 4.26	51.2 ± 4.84

**Table 3:** Cell and protein analyses of induced sputum 18 hr after 1D and 4D exposure to O_3_ [median (25–75% range)].

Analyte	1D	4D
Total leukocytes (cells × 10^4^/mL)	42.7 (23.2–60.6)	45.8 (31.3–71.0)
Neutrophils (%)	56.7 (52.2–74.1)	72.5 (66.3–79.7)*
Macrophages (%)	40.7 (25.4–43.0)	27.0 (18.5–31.9)**
Lymphocytes (%)	1.3 (0.8–4.2)	1.8 (0.2–2.3)
Eosinophils (%)	0.0 (0.0–0.8)	0.0 (0.0–1.2)
Total protein (mg/mL)	908 (624–1,016)	872 (583–976)
LDH (U/L)	78 (64–123)	90 (63–118)
Fibrinogen (ng/mL)	982 (439–2,478)	1,567 (441–3,213)
IL-6 (pg/mL)	73 (15–106)	42 (27–105)
IL-8 (pg/mL)	2,936 (738–7,025)	2,972 (937–4,228)

*p = 0.02 versus 1D neutrophils (%) (Wilcoxon signed rank test);

**p = 0.027 versus 1D macrophages (%) (Wilcoxon signed rank test).
